# Enhancing the biocatalytic manufacture of the key intermediate of atorvastatin by focused directed evolution of halohydrin dehalogenase

**DOI:** 10.1038/srep42064

**Published:** 2017-02-06

**Authors:** Yu Luo, Yangzi Chen, Hongmin Ma, ZhenHua Tian, Yeqi Zhang, Jian Zhang

**Affiliations:** 1 Department of Pathophysiology, Key Laboratory of Cell Differentiation and Apoptosis of Chinese Ministry of Education, Shanghai Jiao-Tong University School of Medicine (SJTU-SM), Shanghai 200025, China; 2 Abiochem Co. LTD, Shanghai, China; 3 Key Laboratory of Combinational Biosynthesis and Drug Discovery, Ministry of Education, Wuhan University School of Pharmaceutical Sciences, 185 Donghu Road, Wuhan 430071, China

## Abstract

Halohydrin dehalogenases (HHDHs) are biocatalytically interesting enzymes due to their ability to form C-C, C-N, C-O, and C-S bonds. One of most important application of HHDH was the protein engineering of HheC (halohydrin dehalogenase from *Agrobacterium radiobacter* AD1) for the industrial manufacturing of ethyl (*R*)-4-cyano-3-hydroxybutanoate (HN), a key chiral synthon of a cholesterol-lowering drug of atorvastatin. During our development of an alternative, more efficient and economic route for chemo-enzymatic preparation of the intermediate of atorvastatin, we found that the HheC2360 previously reported for HN manufacture, had insufficient activity for the cyanolysis production of *tert*-butyl (*3 R,5 S*)-6-cyano-3,5-dihydroxyhexanoate (A7). Herein, we present the focused directed evolution of HheC2360 with higher activity and enhanced biocatalytic performance using active site mutagenesis. Through docking of the product, A7, into the crystal structure of HheC2360, 6 residues was selected for combined active sites testing (CASTing). After library screening, the variant V84G/W86F was identified to have a 15- fold increase in activity. Time course analysis of the cyanolysis reaction catalyzed by this variant, showed 2- fold increase in space time productivity compared with HheC2360. These results demonstrate the applicability of the variant V84G/W86F as a biocatalyst for the efficient and practical production of atorvastatin intermediate.

Halohydrin dehalogenases (HHDHs, EC 4.5.1.X), also known as halohydrin epoxidases or halohydrin hydrogen-halide-lyases, are microbial enzymes that catalyze the reversible dehalogenation of β-haloalcohols with stereo-selective formation of epoxides. Naturally, these enzymes are involved in the degradation of halogenated pollutants such as epichlorohydrin^1^. Due to the promiscuous and irreversible ring-opening activities of these enzymes undergoing nucleophilic attack by a diverse range of anionic nucleophiles such as CN-, NO2-, N3-, SCN-, HCOO-, and OCN-, many β-functionalized alcohols could be obtained, which could then be further converted into synthons for synthesis of fine chemicals and pharmaceutic intermediates^2^,^3^.

Therefore, HHDHs are becoming remarkable biocatalysts in synthetic chemistry^4^,^5^. For example, as only five different HHDHs had been employed for C-C, C-N, C-O, and C-S bonds formation over the past 20 years^4^,^6^, a motif-based enzyme identification approach was recently adopted, resulting in 37 new HHDH genes with 37% to 60% sequence identities to the known HHDHs^7^. This was achieved, via identifying the Ser-Tyr-Arg catalytic triad of HHDHs in combination with the HHDH -specific anion binding Phe or Tyr residue of the homologous proteins. Among these HHDHs, 17 representatives from six phylogenetic subtypes were well characterized, several of which exhibited broad substrate scopes and high activities, and their stereopreference was found to correlate with the phylogenetic placing into the HHDH enzyme family subtypes^8^. Among all HHDHs mentioned above, the halohydrin dehalogenase from *Agrobacterium radiobacter* AD1, named HheC, turns out to be the most biochemically, mechanistically and structurally investigated HHDH^9^,^10^. HheC was engineered to enhance the cyanolysis activity through 18 rounds of directed evolution driven by protein sequence activity relationships^11^. The engineered HHDH, named HheC2360, which exhibited twofold improvement in activity and 8 °C improvement in apparent T_50_^10^ value^12^, was then industrially applied for the manufacture of ethyl (*R*)-4-cyano-3-hydroxybutanoate (**HN**), a key chiral building block of atorvastatin^13^.

Atorvastatin is a salable cholesterol-lowering drug with worldwide annual sales exceeding 10 billion dollars^14^,^15^. Its industrial manufacture is thus attractive, and *tert* -butyl (*4 R,6 R*)-6-cyanomethyl-2,2-dimethyl-1,3-dioxane-4-acetate (**A8**) serves as the key synthetic intermediate in most routes (Fig. 1)^14^,^15^. Using HheC2360 mentioned above as well as mutants of ketoreductase and glucose dehydrogenase, HN was obtained^13^. The downstream processes of this KRED route (Fig. 1) employed harsh chemical conditions, such as the Claisen condensation mediated by lithium diisopropylamide (LDA) at −78 °C^14^,^15^. The third generation of the synthetic route involved asymmetric aldol addition of chloroacetaldehyde with two acetaldehyde catalyzed by deoxyribose phosphate aldolase (DERA)^16^. We previously extended this route for the synthesis of A8 by GDH-catalyzed oxidation of the lactol to lactone^17^, followed by several chemical synthetic steps (Fig. 1). However, the cyanolysis of *tert*-butyl (*3 R,5 S*)-6-chloro-3,5-dihydroxyhexanoate (**D3**) appeared to be rather challenging. The chemical route employed alkaline conditions, during which the substrate D3 self-hydrolyzed rapidly, leading to a yield lower than 50%. Meanwhile, the enzymatic route using HheC2360 during which the netural pH caused no hydrolysis of the ester exhibited low activity towards D3 compared with ethyl 4-chloroacetoacetate, and thus higher concentration of the enzyme was needed which made the downstream separation rather difficult.

Herein, we set out to perform the structure- guided protein engineering of HheC to enhance its biocatalytic performance. Through docking the product *tert*-butyl (*3 R,5 R*)-6-cyano-3,5-dihydroxyhexanoate (**A7**) into the HheC crystal structure (PDB code: 4IXT)^12^, six residues were selected and divided into three groups based on the possibilities of cooperative effects. By screening of the three resulting libraries, a mutant, V84G/W86F, with 15-fold increased activity was obtained, which improves the volumetric productivity of the cyanation process 2-fold. Thus, this work has exhibited feasibility for the practical production of the atorvastatin intermediate, A8.

## Results

### Docking A7 into HheC2360

To identify the key residues interacting with the substrate D3, the compound A7 was docked into the crystal structure of HheC2360 (PDB code: 4IXT)^12^ with the co-crystalled HN removed and then optimized by selection of the conformation with minimum RMSD of the terminal hydroxypropionitrile motif, since the co-crystalled HN serves as enzymatic product of HheC2360. With the HheC2360/A7 complex model, the residues V84, W86, W139, L142, Y186 and Y187 were found to be located in the 5 Å radius of the compound, and these residues were classified as three groups (V84-W86, W139-L142, Y186-Y187) based on the possibilities of cooperative effects for combinational active-site saturation testing (CASTing), a widely used method for increasing the activities of biocatalysts^18^.

### Mutant libraries screening

Mutant libraries of the combined active sites were constructed, and a high throughput screening strategy was adopted, via determining the concentration of chloride ion formed during the biocatalytic dehalogenation process based on the red complex (iron(III)/thiocyanate) with a characteristic absorbance at 460 nm^19^. For the V84-W86 library, 14 beneficial mutants were identified with improvement of biocatalytic efficiency after a comprehensive screen of 3072 colonies to cover most of the possibility^20^. The activities of the mutants were further determined and listed in Fig. 2. Sequence analysis of these mutants demonstrated that all had contained the mutation of W86. The three single mutants, W86V, W86L, and W86I, exhibited roughly 5- folds higher activity. Most of the combined mutants showed similar or lower activity than the single mutants, except for V84G/W86V and V84G/W86F, which respectively gave 9- and 15- fold higher activity than Hhec2360.

### Determination of the kinetic constants of the HHDHs

The kinetic constants, of the purified HheC2360, W86V, V84G/W86V and V84G/W86F, were determined by fitting the activities of these enzymes towards various concentrations of D3 with the Michaelis –Menten equation and listed in Table 1. All of the mutants mentioned above exhibited increased *k*_*cat*_ values and decreased *K*_*m*_ values. W86V showed a 2- fold decrease in *K*_*m*_ and almost 2.5- fold increase in *k*_*cat*_, and the combination of an additional V84G caused a further decrease in *K*_*m*_ and increase in *k*_*cat*_. Although the V84G/W86F mutant exhibited a similar *k*_*cat*_, the still smaller *K*_*m*_ value granted it 1.85- fold higher enzymatic efficiency (*k*_*cat*_/*K*_*m*_, 390 s^−1^ mM^−1^) compared to the V84G/W86V mutant (*k*_*cat*_/*K*_*m*_, 211 s^−1^ mM^−1^), and thus 16- fold increased catalytic efficiency was achieved over HheC2360 (*k*_*cat*_/*K*_*m*_, 24.1 s^−1^ mM^−1^).

### Comparative bioconversion performance

To verify the biocatalytic efficiency of the variant, the crude extract of both HheC2360 and the V84G/W86F mutant overexpressed in *E. coli* BL21 (DE3) were used directly as cost-effective biocatalysts^21^ for the cyanolysis of D3 to A7. To eliminate the protein concentration difference, the cell lysate were diluted to the same concentrations (20 mg/mL). As shown in Fig. 3, compared to the template HheC2360, the mutant showed a similar rate of forming the epoxide intermediate, but a 3- fold faster rate of forming the product A7 and a 1.8- fold increased rate of consuming the substrate D3. The phenomenon that bioconversion rates of the cell lysates of the two biocatalysts were not fully consisted with the difference between their initial activities, was also observed in other engineered biocatalysts^22^. Full conversion catalyzed by the mutant was achieved in 24 h, and an additional 20 h was required for HheC2360. The results demonstrated a two-fold elevated bioconversion rate in our variant, which shows promising the applicability of our mutant for the cost-effective manufacture of atorvastatin intermediate.

## Discussion

The discovery, application and engineering of HHDHs have developed rapidly during the past few years due to their practical application in the synthesis of optically active epoxides and β-functional alcohols^23^,^24^,^25^,^26^,^27^. However, even engineered biocatalysts for manufacture of one certain fine chemical could not fully meet the industrial requirements for synthesis of another product and thus more engineering efforts are needed. For example, the engineered ketoreductase for production of (*S*)-1-(2,6-dichloro-3-fluorophenyl)-ethanol was further evolved for manufacture of montelukast, which in turn was a starting point for the duloxetine ketoreductase^14^. The structural variations of substrates may abolish recognition and interactions between a certain substrate and the enzyme designed for it. Besides, as the size of the substitutional group varies, the binding pocket suitable for the desired subtrate cannot meet other substrates. Therefore, further evolution was necessary to improve the activity for these substrates. In this context, as the engineered HHDH for preparation of HN did not exhibit enough activity towards the substrate D3 in our synthetic route to produce the statin intermediate, focused protein engineering of the active sites to increase enzymatic activity was conducted in this study.

To ensure the accuracy of the model of the docked product A7 with the previously reported HheC2360, the conformation with minimal RMSD of the β-hydroxynitrile motif of HN and A7 was selected. By selecting residues in proximity of the docked product and omitting those pointing outward the binding pocket, 14 amino acids, namely F12, V84, W86, S132, A134, W139, L142, Y145, R149, N176, Y186, Y187, W192 and W201 were identified (Fig. 4). Among these residues, S132, Y145 and R149 served as active sites (Fig. 4b), and the aromatic rings of F12, Y186 and Y187 were believed to interact with anions such as halides and cyano (Fig. 4c). Y186, Y187 were also close to the large *t*-butyl motif of A7 and we thus anticipated that mutation of the two residues could create more room for it. T134A in HheC2360 (HheC2360 bears the T134A mutation) was believed to abolish the hydrogen bonding interaction with the catalytic triad S132, resulting in consequent increase in enzymatic activity^12^,^28^. In the modeled structure, an additional hydrogen bond could be formed between N176 and the additional hydroxyl group adjacent to the hydroxynitrile of A7 (Fig. 4b). The conserved W192 located in a hydrophobic cavity at the interface of two subunits was believed to stabilize the tetrameric enzyme assembly of HHDHs and it was reported that mutagenesis of W192F resulted in total loss of the enzymatic activity possibly due to the destruction of the interactions within the cavity and the accompanying change of the oligomeric state^12^,^29^. H201W was believed to enhance the oligomeric stability by extending the aromatic interaction network in HheC2360^12^. Therefore, the remaining residues in the vicinity of the substrate, namely V84, W86, L142, W139, Y186 and Y187, were then chosen for combinatorial active-site saturation testing (CASTing)^18^ to enhance the enzymatic activity.

P84V caused shape changement of the active site; F86W affected the rotational freedom of W139, which created room for an aliphatic substrate^12^. However, for A7, strong steric hindrance between V84, W86 in HheC2360 and the extended *t*- butyl group of the substrate was observed owing to the 3.3 Å distance between W86 and A7 and 3.5 Å distance of V84 compared to 3.8 Å of Y187, 4.0 Å of Y186, 4.2 Å of W139, 4.6 Å of W192, 5.5 Å of L142, and 6.2 Å of W201. Therefore, mutation of the closest V84 and W86 residues could more easily create room to fully accommodate the substrate and thus improve the activity. Experimentally, single mutations of W86 resulted in improved activity. For example, all of the single mutants, W86V, W86L, and W86I had all with 5.0- fold activity improvement. Mutations of both V84 and W86 to hydrophobic residues were rather preferred as most of the mutants were V, I, L, F, P, C, M, and G, which may be attributed to the hydrophobic nature of the t- butyl moiety. One of the exceptions were V84L/W86R showing 1.5- fold increase in activity, but this is lower than the V84L/W86L (5.0- fold) and V84L/W86I (3.0- fold). Similarly, mutations of V84 into hydrophilic S and T were unfavorable as the activities of V84S/W86V (3.0- fold) and V84T/W86I (3.3- fold) were lower than the corresponding W86V, W86L, or W86I mutants (all 5.0- fold), probably due to the unfavorable interaction between the hydrophilic S or T of the residues and the lipophilic *t*- butyl motif of A7. Combination of V84P and W86L, which enlarged the binging pocket, resulted in unaltered activity (5.0- fold). The mutant V84P/W86P even exhibited lower activity (4.0- fold). It was stated that the mutation of P84V in HheC2360 results in Cα positional changes in the loop between A83 and W86. This leads to shape alteration of the active site, and the modified binding pocket is more suitable for binding an aliphatic group than an aromatic ring^12^. Therefore, the V84P mutant obtained has likely changed the binding pocket again in a way that the binding pocket turns out to be more suitable for an aromatic ring, which leaded to weaker affinity for our aliphatic substrate. In addition, consindering the tendency of proline to form cis peptides, V84P may cause rotation of the carbonyl group of preceding P83 as shown in Figure S3, which made the binding pocket more hydrophilic and thus led to reduced activity. Unlike proline, the flexibility of glycine residues could make the shape of the active site unchanged. Meanwhile, the smallest volume of glycine residues could confer the binding pocket bigger volume than other amino acids to accommodate large *t*-butyl moiety of the substrate D3 so that this substrate could form the transition state as shown in Fig. 4b more easily. Therefore, with the easing of the enzyme-substrate clash, V84G/W86V exihibited higher activity (9.0- fold) than W86V and V84P/W86L. Mutations of W86 into residues with small side chains, such as P and C, leaded to decreased activities. V84G/W86F exhibited highest activity (15- fold). This phenomenon may be attributed to the aromatic network of W86F, W139, W192 and W201 (Fig. 4c), and that W86F had created enough room for the substrate. The decreasing *K*_*m*_ value of HheC2360, W86V, V84G/W86V and V84G/W86F suggested increasing interaction, while the rising *k*_*cat*_ value implied the increasing room to accommodate the substrate. From the models shown in Fig. 4d and e, enlarged binding pocket of V84G/W86F compared with HheC2360 could be observed. The similar *k*_*cat*_ value of V84G/W86V and V84G/W86F indicated that W86F had granted the enzyme enough room and higher affinity, which is consistent with our assumption.

The minimum distance between the compound and L142 was determined as 5.5 Å, which is rather long for receptor –ligand interaction. Despite the closer distance (4.6 Å), W139 was believed to be involved in the aromatic system^12^, and both W139F^29^ and W139C^30^ resulted in reduced activity due to the dozens fold increased *Km* value^29^,^30^. The aromatic rings of both Y186 and Y187 were related to anion binding, and thus both exhibited only Y and F variability by sequence alignment. Y186 in HheC2360 stabilized a rare non-proline cis-peptide bond compared to F186 in WT HheC^12^. Y187F in HheC resulted in improved elimination activity of halides but reduced cyanolysis activity^31^. Therefore, it is not surprising that screening of the W139/L142 and Y186/Y187 libraries failed to obtain mutants with increased activity.

**Figure 1:**
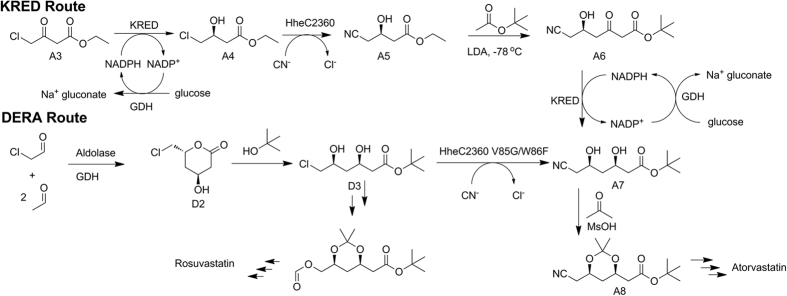
The synthetic routes to atorvastatin intermediate. The KRED Route employed KRED-GDH catalyzed ketoreduction, cyanation, claisen condensation, another ketoreduction and isopropylidenation, while the DERA Route adopted DERA catalyzed aldol reaction, esterification, cyanolysis, and isopropylidenation.

**Figure 2:**
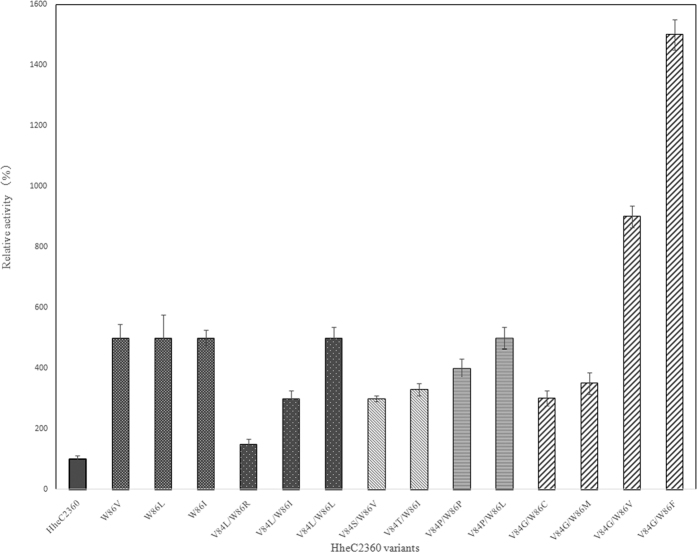
Relative activities of HheC2360 mutants. The relative activities was determined by the release of chloride ion. The activities of HheC2360 (207 U/mg) was used as the control (100%).

**Figure 3:**
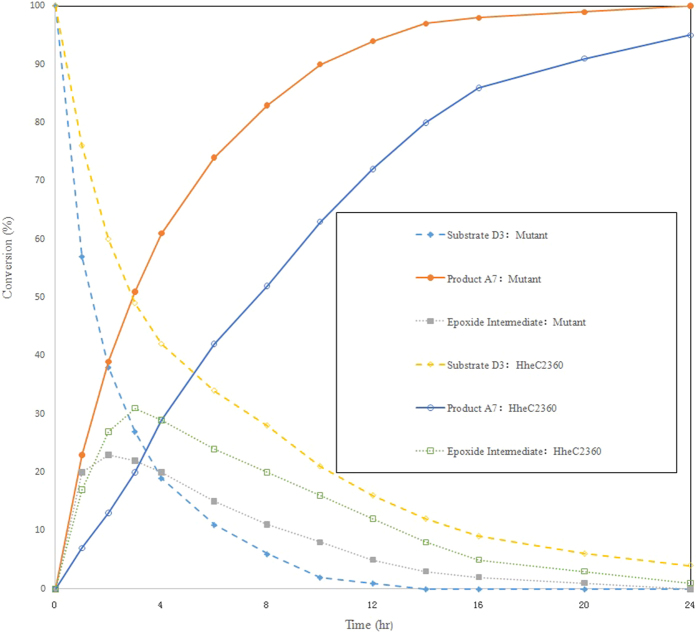
Time Course of HheC2360 (*hollow*) and the V84G/W86F mutant (*solid*) catalyzed cyanolysis. *Symbols*: comsuming of substrate D3 (*diamonds*), formation of product A7 (*circles*), and apparence and disapparence of the epoxide intermediate (*squares*).

**Figure 4:**
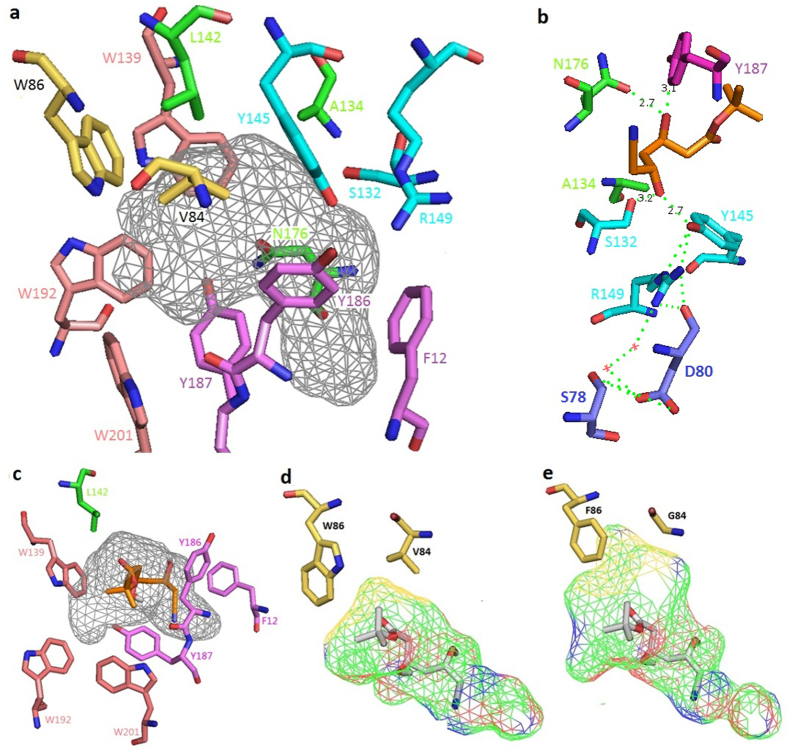
Binding poses of the product A7 in HheC2360 and the V84G/W86F variant. (**a**) An overview of the binding pocket of HheC2360. The catalytic triad S132-Y145-R148 were painted with *cyan*. The 84 and 86 positions of the two biocatalysts were painted with*yellow*. The anion binding F12, Y186 and The anion binding F12, Y186 and 187 were painted with *violet*. The aromatic ring system W139, W192 and W201 were painted with *salmon*. The other residues were painted with *green*. (**b**) Hydrogen bonds between A7 and the catalytic S132-Y145 as well as Y186, N176 and the hydrogen bonding network at the active site. (**c**) Details of the anion binding F12, Y186, Y187, the aromatic ring system W139, W192, W201 and the adjacent L142. (**d**) Binding pocket of HheC2360. (**e**) Binding pocket of V84G/W86F.

The engineered HHDH with mutations of V84G and W86F was then subjected to the cyanolysis reaction. Its 2- fold increase in productivity could cut the costs of the process places, reaction kettle, energy use as well as human resources, and thus make the process more economically feasible, and is significant for industrial applications. A straightforward chemo-enzymatic synthetic route, involving DERA-GDH catalyzed asymmetric preparation of the statin lactone (*4 S,6 R*)-6-(chloromethyl)-4-hydroxy-oxan-2-one (**D2**), chemical esterification of **D2** to obtain its *t*- butyl ester **D3**, cyanolysis reaction catalyzed by our mutant V84G/W86F to get **A7**, and p-toluenesulfonic acid catalyzed isopropylidenation to obtain the disired product **A8**, is well established (Fig. 1). Meanwhile, the well-established KRED route involved KRED-GDH catalyzed asymmetric reduction of ethyl 4-chloro-3-oxobutanoate (**A3**) to obtain ethyl (*S*)-4-chloro-3-hydroxylbutanoate (**A4**), followed by HHDH catalyzed cyanolysis reaction to obtain HN, Claisen Condensation of HN and *t*-BuOAc to obtain *tert*-butyl (*R*)-6-cyano-5-hydroxy-3-oxohexanoate (**A6**), another KRED-GDH catalyzed asymmetric reduction to get **A7** (Fig. 1). Compared to the well-established KRED route that employs LDA mediated Claisen Condensation (Fig. 1), which requires low temperature and anhydrous conditions, this route adopted esterification as the chemical step, which is among the simplest reactions in synthetic chemistry. Of note, esterification is also required for the KRED Route, since *t*-BuOAc was used as a starting material for Claisen Condensation. All of the differing materials, such as acetaldehyde, chloroacetaldehyde, and catalytic amount of sulphuric acid, were among the cheapest bulk chemicals. In addition to its rather inexpensive price, the advantage of adopting chloracetaldehyde as the starting material of DERA-catalyzed aldol condensation, lies on the fact that the product could be further transformed to the intermediate for rosuvastatin, and this enzymatic substitution of chloride ion by formate ion instead of cyano ion catalyzed by HHDH is still in progress. Here we have established a solid basis for the DERA route studied here, which has great potential for industrial applications.

In conclusion, we have engineered HheC2360 by mutagenesis of the residues in the active site pocket revealed by computional docking analysis. After library screening of combined residues, the mutant V84G/W86F was identified with 15- fold increased activity. Determination of the enzymatic kinetic constants disclosed a 5- fold decreased *K*_m_ value and a 3.3- fold increased *k*_*cat*_ value, which suggested improved affinity and enlarged binding space for the substrate. Time-course analysis of the biocatalytic cyanolysis catalyzed by V84G/W86F showed a 2- fold increase in productivity compared to HheC2360, making the process more economically feasible. Thus we have established the chemo-enzymatic route for atorvastatin intermediate.

## Materials and Methods

### Bacterial strains, plasmids, and reagents

*E. coli* DH5α was used for library construction and subsequent screening, while *E. coli* BL21 (DE3) were used for protein expression. Plasmid pWF1 with tac promotor constructed in our laboratory, was used for library construction, and pET28a (Novagen) was employed as an expression vector. All chemicals and biochemicals were from standard commercial sources.

### Cloning and mutagenesis

The *hheC2360* gene was codon optimized for expression in *E. coli* based on the amino acid sequence of the halohydrin dehalogenase under the PDB code of 4IXT^11^,^12^, synthesized and then cloned into the *Spe*I and *Xho*I sites of the pWF-1 to yield pWF-HHDH (Figure S1). Mutant libraries of residues 84–86 as well as 139–142 and 186–187 were constructed by plasmid PCR using primers listed in Table S1 according to the QuikChange^TM^ protocol^32^. The PCR products were digested with *Dpn*I, followed by transformation into JM109 competent cell, and the transformants were plated on Luria Bertani (LB) agar plates with 50 μg/ml ampicillin. The cells were cultured for 16 hours in 96 well plates with 1 ml LB supplemented with 50 μg/ml ampicillin, and proetin expression was induced by addition of 0.2 mM IPTG. The cells were cultured at 25 °C for another 12 h and collected by centrifugation at 3,500 rpm for 10 min at 4 °C. The recovered cells were suspended in lysis buffer (20 mM Tris, 200 mM Na_2_SO_4_, 1 mg/mL lysozyme, pH 7.5) and incubated at 37 °C for 1 h. Cell lysate was then obtained through bacterial freeze thaw.

### Enzyme assay, kinetic constants determinatioin and library screening

The colorimetric assay for determination of HHDH activities was performed. Briefly, 1% of the substrate D3 dissolved in 50% methanol (50 μL) was added into the cell lysate or purified enzyme (450 μL, in 50 mM Tris/SO_4_) to initiate the reactions and incubated at 30 °C for 30 min. After adding saturated Hg(SCN)_2_ in ethanol (200 μL) and 60 g/L of FeNH_4_(SO_4_)_2_.12H_2_O in 1 M HNO_3_ (1800 μL), the released chloride ion was assayed spectrophotometrically by monitoring the absorbance at 460 nm and calculated against the standard curve of sodium chloride. One unit of enzyme activity was defined as the amount of enzyme catalyzing the formation of 1 μmol chloride ion per minute. To calculate the specific activities, the protein concentration was determined using Bradford assay. The activities of HHDHs were calculated using the equation below:


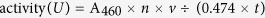


where *n* is the dilution factor of enzyme; *v* is the volume of reaction, in mL (0.45); *t* is the reaction time in minutes (30). 0.474 is the absorbance coefficiency of chloride, using sodium chloride as standard under the equal assay condition.

By varying the substrate concentrations of D3 from 0.05 mM, 0.1 mM, 0.2 mM, 0.5 mM, 1.0 mM, 2.0 mM, to 5.0 mM, the initial velocities were fitted with Michaelis-Menten equation to obtain the kinetic parameters (*k*_*cat*_ and *V*_*max*_).

Library screening was performed at 250 μL of the final product by a microplate reader. Before the assay, the OD_600_ of the suspended cells was measured by the reader to estimate the protein concentration as reported^30^, since all of the HHDH shares similar expression levels (Figure S2).

### Protein expression and purification

The wild type and the mutated HHDH was digested with *Nde*I and *Xho*I, and the resulting fragment was then cloned into the same sites of pET28a (+), followed by transformation into *E. coli* BL21 (DE3) cells. The recombinant *E. coli* was cultivated at 37 °C in LB medium supplemented with kanamycin (50 μg mL^−1^). When OD_600_ reached approximately 0.8, the enzyme expression was induced by IPTG (final concentration = 0.2 mM) and the cells were further cultured at 16 °C for 18 h. Cells were harvested by centrifugation (8,800 rpm, 5 min, 4 °C), washed with lysis buffer, and resuspended in proper volume of lysis buffer (5 mL/g wet cells). The cell suspension was subjected to ultrasonic cell disruption (600 W, 10 seconds per minute for 20 minutes) to obtain the cell lysate. Purification of the wild type and the mutated HHDH were carried out using Ni-IDA sepharose (50% suspension, Qiagen Inc.) and elution buffer was optimized as 150 mM imidazole, 20 mM Tris-H_2_SO_4_, 200 mM Na_2_SO_4_, pH 7.5. Buffer change into 20 mM Tris-H_2_SO_4_ buffer (pH 7.5) containing 100 mM Na_2_SO_4_ was carried out using centrifugal filters (Amicon Ultra, 30 K).

### Preparation of substrate D3

**D2** was biocatalytically synthesized according to previous publications^15^,^16^. Briefly, 150 mM of chloroacetaldehyde and 300 mM of acetaldehyde were pumped into 20% of the recombinant *E. coli* cells overexpressing DERA and a PQQ-dependent glucose dehydrogenase from *E. coli* (**GDH**) at the presence of 2 μM of pyrroloquinoline quinone (**PQQ**) and 10 mM of MgCl_2_. During the reaction, pH was maintained at 7.0 automatically and air flow at 0.5 L/min. At the end of the reaction determined by HPLC^17^, the reaction product was extracted twice with 2 volume of ethyl acetate each after adding Na_2_SO_4_ to 200 g/L. The organic phase was dried over MgSO_4_ and then evaporated to yield D2 as yellow-brownish oil.

H_2_SO_4_ (98%, 15 ml) was added dropwise to tertiary butanol (100 ml) at 0–4 °C. After adding D2 (16.5 g), the solution was refluxed with agitation for 4 h and then washed with saturated Na_2_CO_3_ to a neutral pH. After removal of *t*-BuOH by rotary evaporation, the product was extracted with CH_2_Cl_2_. The organic layer was dried over MgSO_4_ and concentrated to afford D3 as off-white solid.

### Enzymatic cyanolysis of D3 to A7

The protein concentrations of cell lysate of the recombinant *E. coli* cells overexpression HheC2360 or its mutant were diluted to 20 mg/ml with KPB buffer (50 mM, pH 7.0). After adding the substrate D3 (8.0 g) to the cell lysate (50 mL), 10% sodium cyanide solution (20 mL, 1.2 eq) was added dropwise. The reaction was allowed for 24 h at 45 °C when pH was adjusted to 7.0 automatically with 1 M H_2_SO_4_. During the reaction, periodic sampling of the mixture was carried out, followed by HPLC analysis of substrate D3, the epoxide intermediate, and the product A7. At the end of the reaction determined by HPLC analysis, the product was extracted three times with EtOAc^33^. After being dried over MgSO_4_, the organic layer was evaporated to obtain crude A7 as a yellow oil (7.06 g, 92% yield). The HPLC analysis was perform on a Agilent 1100 whcih equipped with Eclipse C18 (4.6 × 250 mm, particle size 3.5 μm), and was eluted with mobile phase A (water supplemented with 0.1% trifluoroacetic acid) and mobile phase B (acetonitrile supplemented with 0.1% trifluoroacetic acid) at the following gradient: 10% phase B at 0 min; 100% phase B at 15 min; 100% phase B at 20 min; 10% phase B at 21 min; 10% phase B at 25 min.

### Chemical conversions of A7 to A8

The crude A7 (10 g) was added into 2,2- dimethoxypropane (30 mL). After adding p-toluenesulfonic acid (0.25 g) to initiate the reaction, the solution was stirred for 30 min at room temperature. Then sodium bicarbonate solution (100 mM, 50 mL) was added, and the mixture was extracted twice with EtOAc (100 mL each). The organic layer was washed over saturated brine, dried over MgSO4, and concentrated to afford crude A8 as a white powder (11.4 g, 92% purity, 79% yield). After recrystallization using petroleum ether as the solvent, 9.9 g of A8 with 99% purity proven by gas chromatography (GC) was obtained. The GC analysis was run on the Shimadzu 2010 gas chromatograph equipped with a DB-5MS column (30 m × 250 μm), with a flame ionization detector and using nitrogen as carrier gas. The oven temperture program was initiated at 130 °C and raise up to 200 °C at 5.0 °C/min; hold 8.0 min and raise to 230 °C at 20.0 °C/min, then hold 16.5 min. ^1^H NMR (300 MHz, CDCl3): δ 1.40(s, 9 H), 1.41(s, 6 H), 1.48–1.73(dd, 2 H), 2.26–2.51(d, 2 H), 2.41–2.66(d, 2 H), 3.8(m, 1 H), 4.43(m, 1 H). MS(ESI)m/z:(M + H) = 270.1

### Product docking

The compound A5 from the crystal structure of HheC bound to A5 (PDB code:4IXT)^12^ was selected and the site sphere was defined with the “From current selection” tool. The compound was further removed and the resulting structure was then applied to docking protocol with the CDOCKER module in Discovery Studio 4.0 as the receptor using A7 as the ligand. The resulting conformations were compared with HN and that with minimum RMSD of the terminal hydroxypropionitrile motif was selected as the final complex. The residues interacting with A7 were identified with selection of the amino acids within 6 Å radius of A7.

### Ethical approval

This article does not contain any studies with human participants or animals performed by any of the authors.

## Additional Information

**How to cite this article**: Luo, Y. *et al*. Enhancing the biocatalytic manufacture of the key intermediate of atorvastatin by focused directed evolution of halohydrin dehalogenase. *Sci. Rep.* **7**, 42064; doi: 10.1038/srep42064 (2017).

**Publisher's note:** Springer Nature remains neutral with regard to jurisdictional claims in published maps and institutional affiliations.

## Supplementary Material

Supplementary Information

## Figures and Tables

**Table 1:** Kinetic constants of HheC2360 variants.

HheC2360 variants	*K*_*m*_ (mM)	*k*_*cat*_ (s^−1^)	*k*_*cat*_*/K*_*m*_ (s^−1^ mM^−1^)
HheC2360	8.5 ± 0.2	204 ± 9	24.1 ± 2.9
W86V	4.3 ± 0.1	517 ± 13	120 ± 5
V84G/W86V	3.1 ± 0.3	653 ± 17	211 ± 12
V84G/W86F	1.7 ± 0.2	671 ± 14	390 ± 15

## References

[b1] Wijngaard A. J. V. D., Janssen D. B. & Witholt B. Degradation of Epichlorohydrin and Halohydrins by Bacterial Cultures Isolated from Freshwater Sediment. Microbiology 135, 2199–2208 (1989).

[b2] Spelberg J. H., Je V. H. V., Tang L., Janssen D. B. & Kellogg R. M. Highly enantioselective and regioselective biocatalytic azidolysis of aromatic epoxides. Organic Letters 3, 41–43 (2001). 11429866 10.1021/ol0067540

[b3] Vries E. J. D. & Janssen D. B. Biocatalytic conversion of epoxides. Current Opinion in Biotechnology 14, 414–420 (2003). 12943851 10.1016/s0958-1669(03)00102-2

[b4] Je V. H. V. . Halohydrin dehalogenases are structurally and mechanistically related to short-chain dehydrogenases/reductases. Journal of Bacteriology 183, 5058–5066 (2001). 11489858 10.1128/JB.183.17.5058-5066.2001 PMC95381

[b5] You Z. Y., Liu Z. Q. & Zheng Y. G. Properties and biotechnological applications of halohydrin dehalogenases: current state and future perspectives. Applied Microbiology & Biotechnology 97, 9–21 (2013). 23111599 10.1007/s00253-012-4523-0

[b6] Yu F., Nakamura T., Mizunashi W. & Watanabe I. Cloning of Two Halohydrin Hydrogen-Halid-Lyase Genes of Corynebacterium sp.Strain N-1074 and Structural Comparison of the Genes and Gene Products. Bioscience Biotechnology & Biochemistry 58, 1451–1457 (1994). 10.1271/bbb.58.1451 7765275

[b7] Schallmey M., Koopmeiners J., Wells E., Wardenga R. & Schallmey A. Expanding the Halohydrin Dehalogenase Enzyme Family: Identification of Novel Enzymes by Database Mining. Applied & Environmental Microbiology 80, 7303–7315 (2014). 25239895 10.1128/AEM.01985-14 PMC4249193

[b8] Koopmeiners J., Halmschlag B., Schallmey M. & Schallmey A. Biochemical and biocatalytic characterization of 17 novel halohydrin dehalogenases. Applied Microbiology & Biotechnology 100, 7517–7527 (2016). 27052376 10.1007/s00253-016-7493-9

[b9] Jong R. M. D. . Structure and mechanism of a bacterial haloalcohol dehalogenase: a new variation of the short-chain dehydrogenase/reductase fold without an NAD(P)H binding site. Embo Journal 22, 4933–4944 (2003). 14517233 10.1093/emboj/cdg479 PMC204463

[b10] Tang L., Vlieg J. E. T. V. H., Spelberg J. H. L., Fraaije M. W. & Janssen D. B. Improved stability of halohydrin dehalogenase from Agrobacterium radiobacter AD1 by replacement of cysteine residues. Enzyme & Microbial Technology 30, 251–258 (2002).

[b11] Fox R. J. . Improving catalytic function by ProSAR-driven enzyme evolution. Nature Biotechnology 25, 338–344 (2007). 10.1038/nbt1286 17322872

[b12] Schallmey A. & Schallmey M. Recent advances on halohydrin dehalogenases-from enzyme identification to novel biocatalytic applications. Applied Microbiology & Biotechnology 100, 7827–7839 (2016). 27502414 10.1007/s00253-016-7750-y PMC4989007

[b13] Ma S. K. A green-by-design biocatalytic process for atorvastatin intermediate. Green Chemistry 12, 81–86 (2010).

[b14] Bornscheuer U. T. . Engineering the third wave of biocatalysis. Nature 485, 185–194 (2012). 22575958 10.1038/nature11117

[b15] Bommarius A. S. Biocatalysis: A Status Report. Annual Review of Chemical and Biomolecular Engineering 6, 319–345 (2015). 10.1146/annurev-chembioeng-061114-123415 26247293

[b16] Ošlaj M. . A highly productive, whole-cell DERA chemoenzymatic process for production of key lactonized side-chain intermediates in statin synthesis. Plos One 8, e62250 (2013). 23667462 10.1371/journal.pone.0062250 PMC3647077

[b17] Vajdič T., Ošlaj M., Kopitar G. & Mrak P. Engineered, highly productive biosynthesis of artificial, lactonized statin side-chain building blocks: The hidden potential of Escherichia coli unleashed. Metabolic Engineering 24, 160–172 (2014). 24858788 10.1016/j.ymben.2014.05.012

[b18] Reetz M. T. Laboratory Evolution of Stereoselective Enzymes: A Prolific Source of Catalysts for Asymmetric Reactions. Angewandte Chemie International Edition 50, 138–174 (2011). 20715024 10.1002/anie.201000826

[b19] Chen S. Y., Yang C. X., Wu J. P., Xu G. & Yang L. R. Multi‐Enzymatic Biosynthesis of Chiral β‐Hydroxy Nitriles through Co‐Expression of Oxidoreductase and Halohydrin Dehalogenase. Advanced Synthesis & Catalysis 355, 3179–3190 (2013).

[b20] Reetz M. T. Biocatalysis in Organic Chemistry and Biotechnology: Past, Present, and Future. Journal of American Chemical Society 135, 12480–12496 (2013). 10.1021/ja405051f 23930719

[b21] Luetz S., Giver L. & Lalonde J. Engineered Enzymes for Chemical Production. Biotechnology & Bioengineering 101, 647–654 (2008). 18814289 10.1002/bit.22077

[b22] Huang L., Ma H. M., Yu H. L. & Xu J. H. Altering the Substrate Specificity of Reductase CgKR1 from Candida glabrata by Protein Engineering for Bioreduction of Aromatic α‐Keto Esters. Advanced Synthesis & Catalysis 356, 1943–1948 (2014).

[b23] Elenkov M. M., Hauer B. & Janssen D. B. Enantioselective Ring Opening of Epoxides with Cyanide Catalyzed by Halohydrin Dehalogenases: A New Approach to Non-Racemic β-Hydroxy Nitriles. Cheminform 37, 579–585 (2006).

[b24] Elenkov M. M., Tang L., Meetsma A., Hauer B. & Janssen D. B. Formation of Enantiopure 5-Substituted Oxazolidinones through Enzyme-Catalysed Kinetic Resolution of Epoxides. Organic Letters 10, 2417–2420 (2008). 18491860 10.1021/ol800698t

[b25] Molinaro C., Guilbault A. A. & Kosjek B. Resolution of 2,2-Disubstituted Epoxides via Biocatalytic Azidolysis. Organic Letters 12, 3772–3775 (2010). 20681610 10.1021/ol101406k

[b26] Tang L., Zhu X., Zheng H., Jiang R. & Majeric E. M. Key residues for controlling enantioselectivity of Halohydrin dehalogenase from Arthrobacter sp. strain AD2, revealed by structure-guided directed evolution. Applied & Environmental Microbiology 78, 2631–2637 (2012). 22327597 10.1128/AEM.06586-11 PMC3318787

[b27] Chen S. Y., He X. J., Wu J. P., Xu G. & Yang L. R. Identification of halohydrin dehalogenase mutants that resist COBE inhibition. Biotechnology & Bioprocess Engineering 19, 26–32 (2014).

[b28] Schallmey M. . A single point mutation enhances hydroxynitrile synthesis by halohydrin dehalogenase. Enzyme & Microbial Technology 70, 50–57 (2015). 25659632 10.1016/j.enzmictec.2014.12.009

[b29] Tang L., van Merode A. E., Lutje Spelberg J. H., Fraaije M. W. & Janssen D. B. Steady-state kinetics and tryptophan fluorescence properties of halohydrin dehalogenase from Agrobacterium radiobacter. Roles of W139 and W249 in the active site and halide-induced conformational change. Biochemistry 42, 14057–14065 (2003). 14636074 10.1021/bi034941a

[b30] Tang L., Li Y. & Wang X. A high-throughput colorimetric assay for screening halohydrin dehalogenase saturation mutagenesis libraries. Journal of Biotechnology 147, 164–168 (2010). 20399816 10.1016/j.jbiotec.2010.04.002

[b31] Tang L. . Improved catalytic properties of halohydrin dehalogenase by modification of the halide-binding site. Biochemistry 44, 6609–6618 (2005). 15850394 10.1021/bi047613z

[b32] Hogrefe H. H., Cline J., Youngblood G. L. & Allen R. M. Creating randomized amino acid libraries with the QuikChange Multi Site-Directed Mutagenesis Kit. Biotechniques 33, 1164–1165 (2002). 10.2144/02335pf01 12449398

[b33] Chen X., Liu Z. Q., Huang J. F., Lin C. P. & Zheng Y. G. Asymmetric synthesis of optically active methyl-2-benzamido-methyl-3-hydroxy-butyrate by robust short-chain alcohol dehydrogenases from Burkholderia gladioli. Chemical Communications 51, 12328–12331 (2015). 26140446 10.1039/c5cc04652a

