# Supplementary material for: Enhancing the biocatalytic manufacture of the key intermediate of atorvastatin by focused directed evolution of halohydrin dehalogenase
Source: Sci Rep. 2017 Feb 6;7:42064. doi: 10.1038/srep42064 (PMC5292711; doi:10.1038/srep42064)

**Supplementary File**

**Enhancing the biocatalytic manufacture of the key intermediate of atorvastatin by focused directed evolution of halohydrin dehalogenase**

Yu Luo^1, 2^, Yangzi Chen^1^, Hongmin Ma^3,*^, Zhen-Hua Tian^2^, Yeqi Zhang^3^, and Jian Zhang^1,*^

^1^ Department of Pathophysiology, Key Laboratory of Cell Differentiation and Apoptosis of Chinese Ministry of Education, Shanghai Jiao-Tong University School of Medicine (SJTU-SM), Shanghai 200025, China

^2^ Abiochem Co. LTD, Shanghai, China

^3^ Key Laboratory of Combinational Biosynthesis and Drug Discovery, Ministry of Education, Wuhan University School of Pharmaceutical Sciences, 185 Donghu Road, Wuhan 430071, China

^*^ Corresponding to M.H. (email: hongminma@whu.edu.cn) and Z.J. (email: [jian.zhang@sjtu.edu.cn](mailto:jian.zhang@sjtu.edu.cn))

Supplementary Table S1. Primes used for library construction.

| Mutant Library | Prime name | Sequence |
| --- | --- | --- |
| V84-W86 | VW84f | 5’-GTTTCTAACGACATCGCTCCGNNKGAANNKCGT  CCGATCGACAAATACGCT-3’ |
|  | VW84r | 5’-AGCGTATTTGTCGATCGGACGMNNTTCMNNCGG  AGCGATGTC GTTAGAAAC-3’ |
| W139-L142 | WL139f | 5’-TCTGCTGCTCCGTTCGGTCCGNNKAAAGAANNK  TCTACCTACTCTTCTGCTCGT-3’ |
|  | WL139r | 5’-ACGAGCAGAAGAGTAGGTAGAMNNTTCTTTMN  NCGGACCGAACGGAGCAGCAGA-3’ |
| Y186-Y187 | YY186f | 5’-CACTCTGGTGACTCTCCGTACNNKNNKCCGTCT  GAACCGTGGAAAACC-3’ |
|  | YY187r | 5’-GGTTTTCCACGGTTCAGACGGMNNMNNGTACG  GAGAGTCACCAGAGTG-3’ |

**Supplementary Figure S1. Schematic presentation of the plasmid pWF-HHDHs for library construction.** pWF-1 is an expression vector which was constructed as following: primers pDXW-F (TTCGGGATCCAAATTCCCCCTTACACGGAG) and pDXW-R (GAAAAGATCAAAGCGCCGGT) were firstly used to amplify a 5.23 kb fragment from plasmid pDXW-8. After digested by BamHI this fragment was self-ligated to generate the plasmid, in which the BamHI site was destroyed by occasion. To replace its coding region, primers pET28-F (ACAGGAAACAGAATTGCCCCTCTAGAAATAATTTTGTTTAAC) together with pET28-R (GCCAAAACAGAAGCTGCAGCAGCCAACTCAGCTTC) were used to amplify the 350 bp coding region of pET28a. This PCR product was treated by Exonuclease III to generate the cohesive ends and ligated with EcoRI-HindIII linearized and Exonuclease III pretreated above plasmid to generate the plasmid pWF-1, in which the coding region of pET28a was inserted between the destroyed EcoRI and HindIII sites.


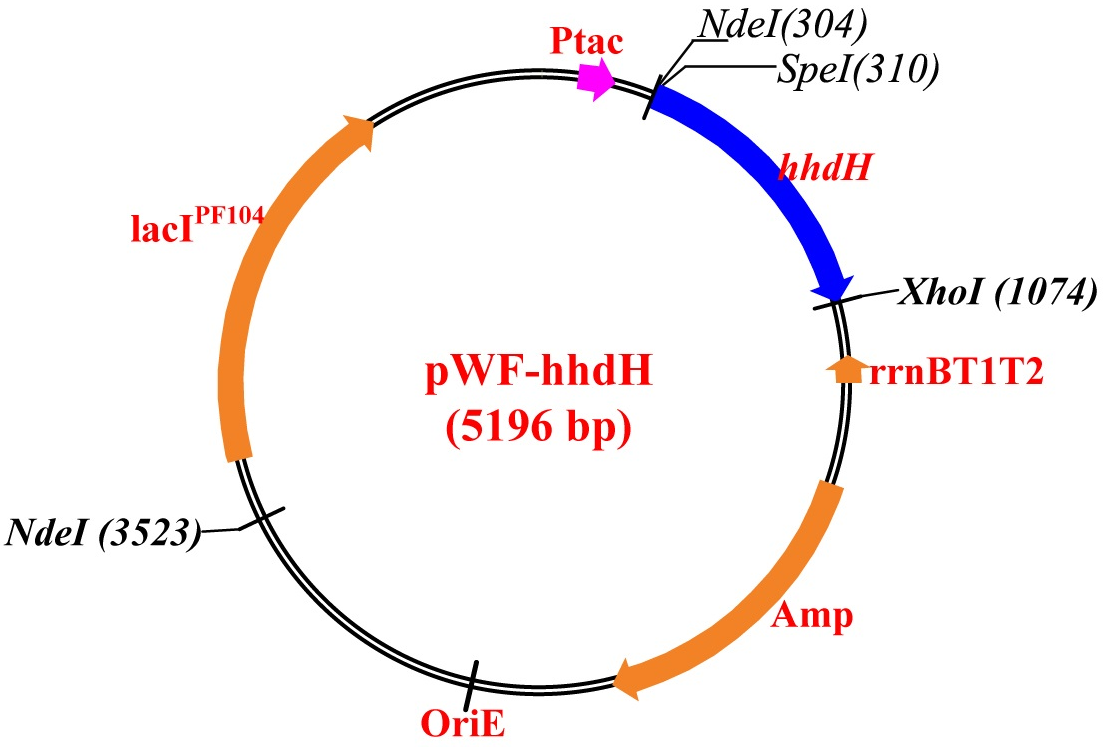


**Supplementary Figure S2. SDS-PAGE analysis of the expression levels of HHDHs expressed in pET28a.** Lane 1, Soluble fraction of cell-free extract of E. coli expression HheC2360. Lane 2, Soluble fraction of cell-free extract of E. coli expression V84G/W86V. Lane 3, Soluble fraction of cell-free extract of E. coli expression V84G/W86F. Lane 4, protein ladder (from top: 170kDa, 130kDa, 100kDa, 70kDa, 55kDa, 40kDa, 30Da, 25kDa, 15kDa, 10kDa). The expression levels estimated by ImageJ of HheC2360, V84G/W86V and V84G/W86F are 23 %, 21 % and 23 %, respectively.


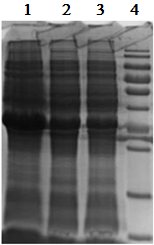


**Supplementary Figure S3. Loops of 82-85 of HheC2360 V84P (a), WT HheC (b) and HheC2360 (c).** The loops of HheC2360 V84P, HheC and HheC2360 as well as the product A7 were painted with *gray*, *cyan*, *green* and *yellow*, respectively.


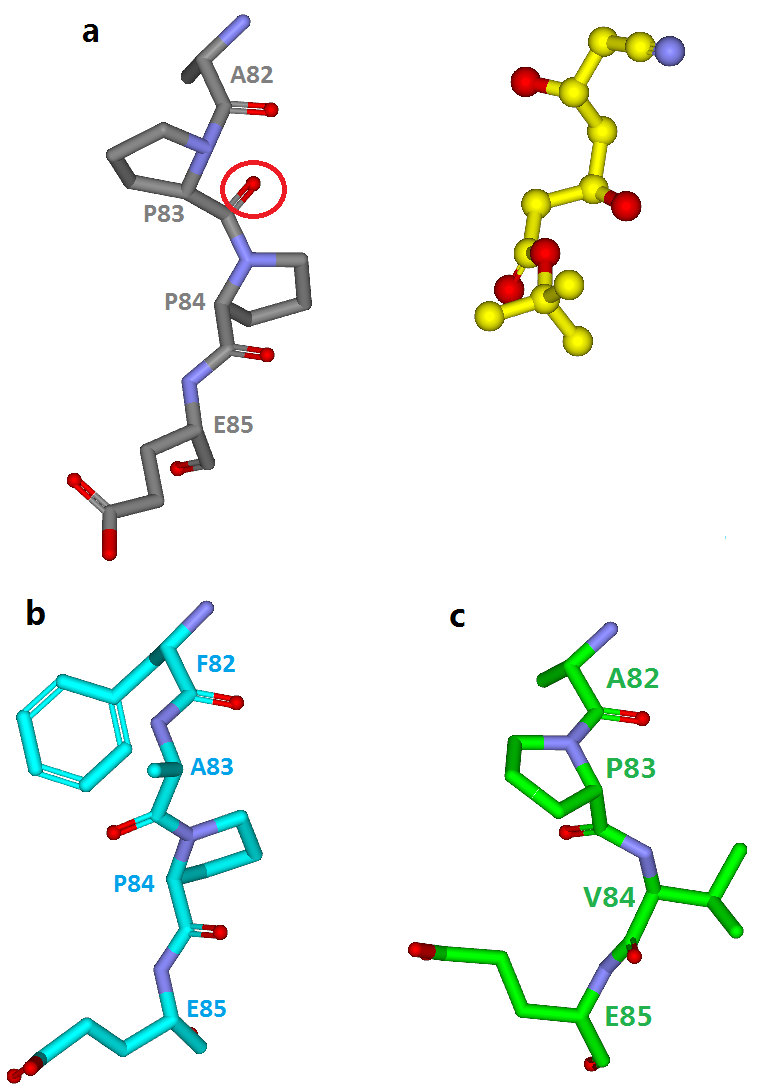


**Supplementary Figure S4. HPLC analysis of the enzymatic cyanation process at 0 h (a), 2 h (b) and 4 h (c).**

**
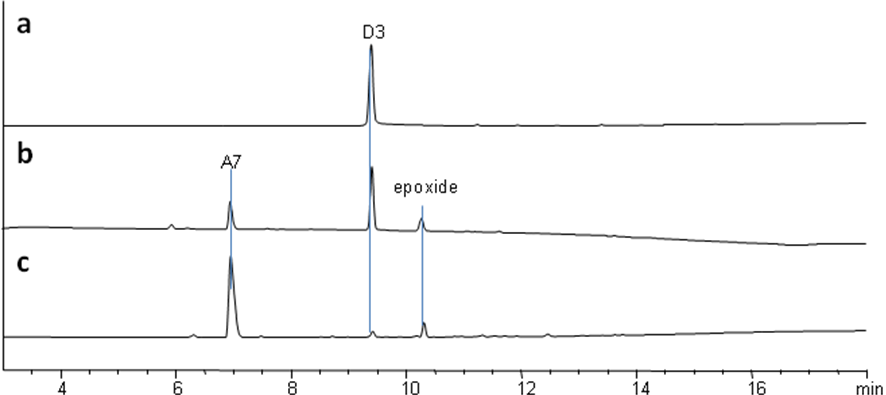
**

**Supplementary Figure S5. GC analysis of A8 standard (a), crude product of chemical isopropylidenation of A7 (b) and the product after recrystallization (c).**


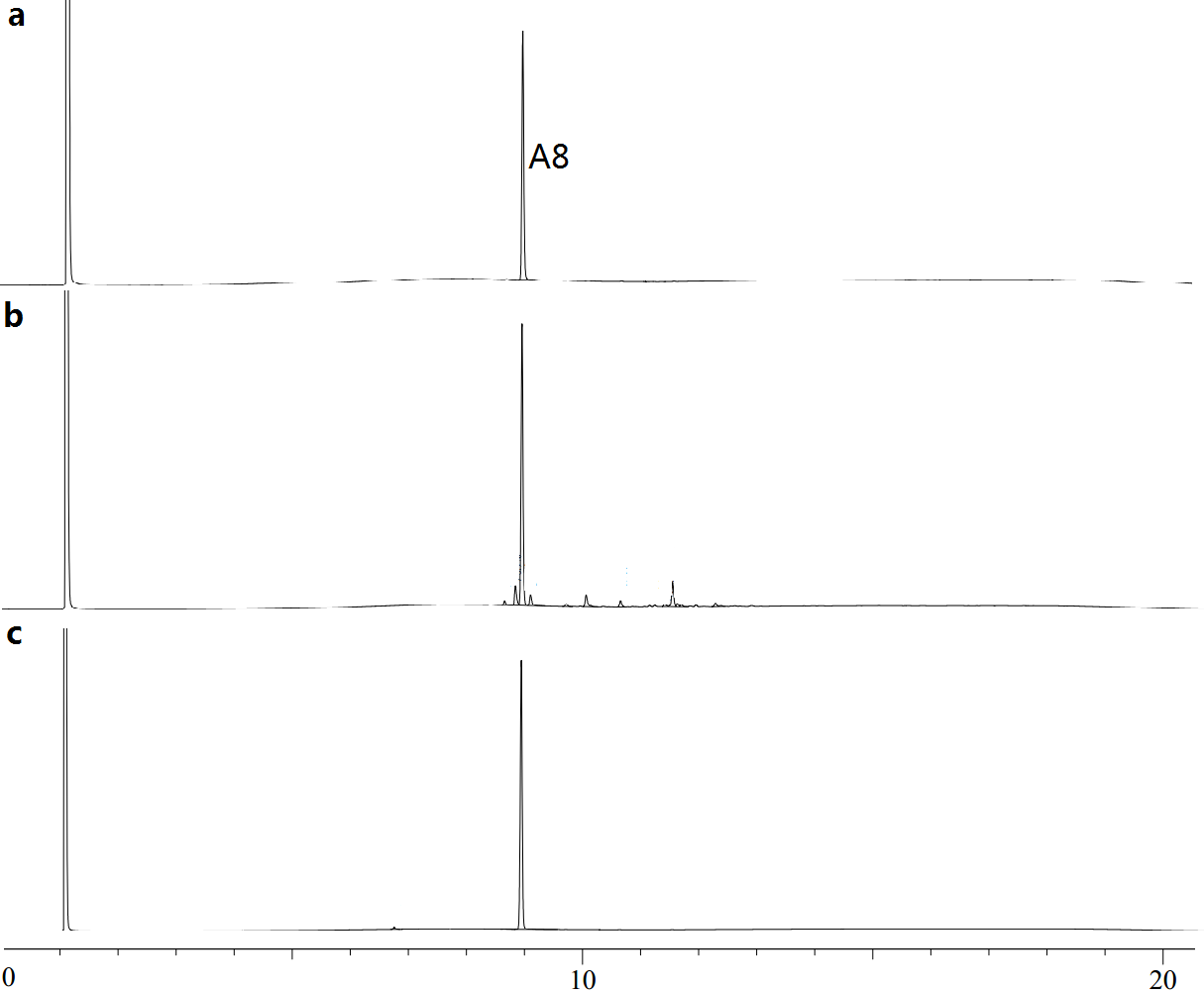

Supplement: Supplementary Information [file srep42064-s1.doc]
